# Real-time adaptive cancellation of TENS feedback artifact on sEMG for prosthesis closed-loop control

**DOI:** 10.3389/fbioe.2024.1492588

**Published:** 2024-11-21

**Authors:** Byungwook Lee, Kyung-Soo Kim, Younggeol Cho

**Affiliations:** ^1^ Department of Mechanical Engineering, Mechatronics Systsems and Control, Korea Advanced Institute of Science and Technology, Deajeon, Republic of Korea; ^2^ HRI^2^ , Istituto Italiano di Tecnologia, Genoa, Italy

**Keywords:** TENS, artifact, prosthetic hand, surface electromyography, adaptive filter, sensory feedback

## Abstract

**Introduction:**

The prosthetic hand has been aimed to restore hand functions by estimating the user’s intention via bio-signal and providing sensory feedback. Surface electromyogram (sEMG) is a widely used signal, and transcutaneous electrical nerve stimulation (TENS) is a promising method for sensory feedback. However, TENS currents can transmit through the skin and interfere as noise with the sEMG signals, referred to as “Artifact,” which degrades the performance of intention estimation.

**Method:**

In this paper, we proposed an adaptive artifact removal method that can cancel artifacts separately across different frequencies and pulse widths of TENS. The modified least-mean-square adaptive filter uses the mean of previous artifacts as reference signals, and compensate using prior information of TENS system. Also temporal separation for artifact discrimination is applied to achieve high artifact removal efficiency. Four sEMG signals—two from flexor digitorum superficialis, flexor carpi ulnaris, extensor carpi ulnaris—was collected to validate signals both offline and online experiments.

**Results and Discussion:**

We validated the filtering performance with twelve participants performing two experiments: artifact cancellation under variable conditions and a real-time hand control simulation called the target reaching experiment (TRE). The result showed that the Signal-to-Noise Ratio (SNR) increased by an average of 10.3dB, and the performance of four TRE indices recovered to the levels similar to those without TENS. The proposed method can significantly improve signal quality via artifact removal in the context of sensory feedback through TENS in prosthetic systems.

## 1 Introduction

Research on bionic hand has been carried out for upper limb amputees to restore dexterity and functioinality in daily life. Accurately estimating user intention is important for the control performance of prosthetic hand, and one of the most widely used bio-signals for this purpose is surface electromyography (sEMG) which is electrical potential measured on skin surface that occur when muscles are activated (Jiang and Farina, 2014). Since sEMG sensors are placed directly on the surface if the target muscle, there is a strong correlation between muscle activation and the sEMG signal obtained (De Luca, 1997; Chowdhury et al., 2013).

In the field of human-robot interaction (HRI), characteristics mentioned above make sEMG signals significantly important, particularly for intention estimation. Usually sEMG used to predict movement of various joints such as the shoulder (Tigrini et al., 2023a; Tigrini et al., 2023b), elbow (Buongiorno et al., 2018), and lower limbs (Dimitrov et al., 2020; Schulte et al., 2022). Additionally, it is utilized in the control of exoskeletons (Kang et al., 2019; Gui et al., 2019), and also for analyzing muscle fatigue (Cifrek et al., 2009; Moniri et al., 2020) which is valuable for the evaluation of other systems. For intent estimation in prosthetic control, sEMG signals are particularly dominant. Numerous studies have focused on classifying predefined hand gestures using sEMG (Parajuli et al., 2019; Samuel et al., 2019; Earley et al., 2016), or an predicting proportional force estimation (Fougner et al., 2012; Hogan, 1976; Nielsen et al., 2010; Cho and Kim, 2020), making it an essential tool in the advancement of prosthetic hand.

In addition to methodologies for precise intent estimation and advancements in prosthetic hardware development, several studies have been conducted to enhance prostheses to make them more human-like. One focus is the sensory feedback strategy that provides the user with information from the prosthetic hand (Sensinger and Dosen, 2020). Unlike natural hands, the prosthetic hand does not inherently provide sensory information such as force, position, or tactile sensations. Prosthetic hand users have emphasized the importance of developing effective feedback methods (Lewis et al., 2012; Biddiss and Chau, 2007) to enhance control performance (Holmes and Spence, 2004). Some studies have reported that such sensory feedback contributes to the improvement of control performance in prosthetic devices in terms of adaptation speed (Earley et al., 2021) or accuracy (D’Anna et al., 2019). To address this need, various sensory feedback methods have been developed, and one of the most widely employed techniques is electro-tactile stimulation using transcutaneous electrical nerve stimulation (TENS). TENS stands out due to its capability to generate multiple sensations by adjusting parameters such as frequency, pulse width (PW), amplitude, and waveform (Szeto and Riso, 2018). Also, it has high spatial efficiency with low sensory overload (Stephens-Fripp et al., 2018).

Several studies have been conducted to utilize the advantages of TENS for sensory feedback of prosthetics. First, strategies for controlling pulse amplitude were proposed to improve grasping performance. A comparison of a method for controlling amplitude according to grip force and a method for providing additional notification at contact and release (Mastinu et al., 2020), a strategy for connecting the angle of the prosthetic to the vibrotactor and matching finger force to amplitude (Vargas et al., 2021) were proposed, and both showed high grasping performance. Also Valle et al. (2018) compared task performance while encoding linear amplitude or frequency modification. Like amplitude change, pulse width is also a parameter that greatly affects the intensity of stimulation. There are results showing that changes in frequency and pulse width lead to changes in the types of human sensation Ara et al. (2014), and Zhang et al. (2022) compared the degree of human sensory recognition according to these two variables. Multi-modal methods have been proposed to transmit different types of TENS by a single electrode to transfer multiple information at the same time (Choi et al., 2017), and a prosthetic application strategy according to multi-modal feedback were validated (Cho et al., 2023). Furthermore, a comparison is made between the closed loop control performance in response to variations in both frequency and pulse width (Dideriksen et al., 2020). Thus, a great deal of research is being done on methods to alter the TENS parameters in order to enhance the control performance of prosthetic hands.

However, both sEMG signal and TENS are in the close frequency bandwidth which is below 500 Hz (Shaw and Bagha, 2012). Because of this, the current of TENS is transmitted through the skin and causes significant noise in the sEMG signals which is referred to as an “Artifact.” To mitigate the interference of artifacts to estimate user intention by sEMG, both physical and software-wise approaches have been researched. Authors in Jiang et al. (2014) tried to adjust physical components such as electrode placement, waveform type, and using concentric electrodes with opposite phases and validated that the artifacts can be reduced. On a software-wise, authors in Hartmann et al. (2014) proposed that signal blanking in the presence of artifacts through signal processing can improve pattern recognition accuracy. A similar strategy was presented in that the recognition performance can be enhanced by time-division interval between the recording windows and stimulation windows (Dosen et al., 2014). However, despite these approaches increasing the estimation performance, the sEMG signal is lost while the feedback is stimulated. Consequently, the estimation phase becomes dependent on the stimulation phase, leading to performance inconsistency, and the stimulation phase itself has inherent limitations.

To overcome this limitation, The models for artifact cancellation through signal processing have been explored. One research utilized empirical mode decomposition and series notch filters (Pilkar et al., 2016). Also, using independent component analysis algorithm to remove EEG artifact on EMG is proposed in various ways such as adding source of EMG (Li et al., 2021) or utilize with wavelet transform and blind source separation (Lu et al., 2024). These methods shows high restoring performance with real sEMG signals, but it can only be applied to a single frequency of TENS and hard to be used in real-time application.

Jiang et al. (2014) proposed an adaptive filter using another reference electrode referred to as a real-time passive electrode, and they showed that the Signal-to-Noise Ratio (SNR) can be increased. In another research (Mendrela et al., 2016), they presented the Sign-sign Least-Mean-Square (LMS) for the cancellation of the artifact in neural signals, and it was validated in *in vivo* conditions. Both models are based on the adaptive filters for artifact removal, however, noise cancellation level regardless of sensor placement and their performance in intention estimation using sEMG require further verification.

In this paper, we propose a modified adaptive filter to separate artifacts caused by sensory feedback from sEMG signals with minimized signal loss. The primary goal of this research is to acquire sEMG signals generated by muscle contractions selectively, and validate the performance in the intention estimation. The artifact cancellation while maintaining the sEMG signal is a challenge, especially when the TENS parameters such as frequency and PW vary continuously during the use of the prosthetic hand. This method has several implications for systems using TENS. First, the proposed method enables the use of a sensory feedback strategy with varying frequency and pulse width. This is because the comparability cost is low and removal is possible in real-time. In addition, while most existing methods mixed clean EMG and artifact to verify the results, this study increased the reliability of the results by using signals containing actual artifacts. Finally, by adding online experiments for real-time implementation and simultaneous use with intention estimation, it was shown that it can be used for prosthetic intention estimation in the future. As the filter should robustly adapt to these parameter variations, the sEMG signals can remain independent, guaranteeing the consistency of the estimation performance. To validate the proposed method, we designed various feedback scenarios (FS) that have different TENS parameters over time. Additionally, real-time performance was validated through the target-reaching experiments (TRE) simulating the manipulation of the prosthetic hand. The main contribution of this research is presenting the adaptive artifact cancellation filter and validating its performance in prosthetic hand control by using restored sEMG. Since the proposed method utilizes only one sEMG signal and is capable of real-time implementation, it can be applied to various fields of human-robot interaction (HRI) requiring signal processing. In particular, it is applicable when providing sensory feedback during the control of fingers, elbows, and exoskeletons through sEMG signals as mentioned above. Through the verification of the method, it is anticipated that prosthetics, traditionally controlled in an open-loop, can be controlled in a closed-loop. Furthermore, the method is expected to be applicable in similar noise-prone situations, such as repetitive occurrences of noise like heartbeats.

## 2 Materials and methods

### 2.1 Artifact in sEMG signal

**FIGURE 1:**
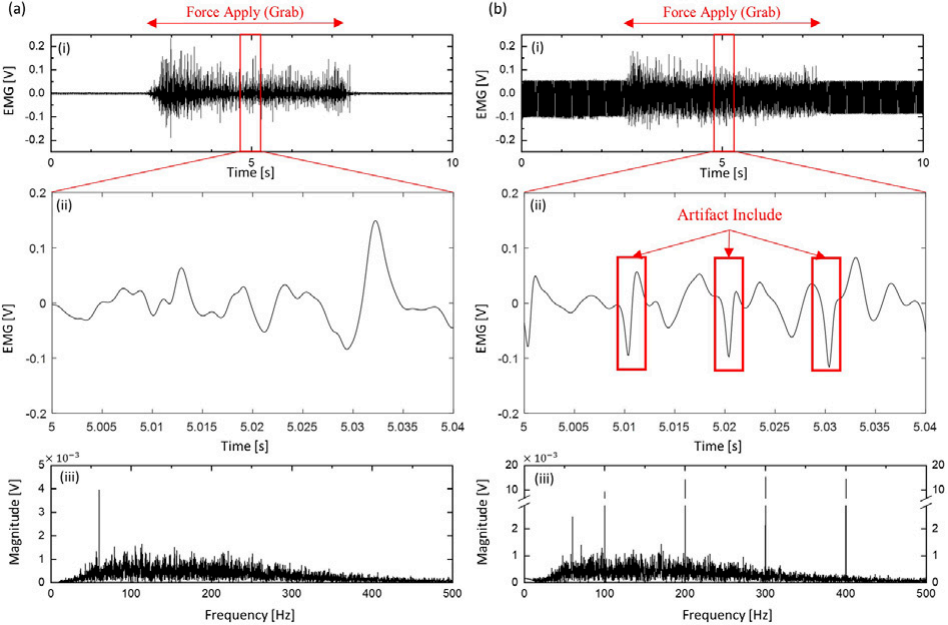
sEMG contaminated by Artifact. **(A)** Without TENS **(B)** 100 Hz, 400us biphasic TENS applied and collected in sEMG sensor (i) Time domain, (ii) Short time period for capture artifact, (iii) Fourier transform of time domain result.

Figure 1 shows the sEMG signal acquired from the same subject and sensor, with and without 100 Hz TENS. By comparing graph (i), it is confirmed that the artifact magnitude level is higher than the muscle signal, making it difficult to discern the muscle signal. In (ii), with a shortened time, the artifact cannot be precisely separated because muscle signals are captured simultaneously. Also, the square biphasic form of TENS is transformed as it passes through the arm, a transformation influenced by factors such as sensor location, arm posture, TENS parameters, etc., Additionally, the duration of the artifact is approximately 3 ms, which varies depending on each channel, PW, etc., but lasts shorter than 10 ms, which is the minimum operation cycle of the developed TENS board. This is because the maximum PW of TENS that a person can generally tolerate is around 500us. In this study, the residual time of the artifact was set to 5 ms (r = 50 samples) to utilize this information in the process described later. The bottom graph in the picture is the FFT result of each time domain graph. Given the TENS with a period of 100 Hz, the 100 Hz harmonic components have large values. Through this, it can be expected that the change in the signal will be significantly dependent on the frequency and PW of the TENS.

### 2.2 Least mean square adaptive filter

**FIGURE 2:**
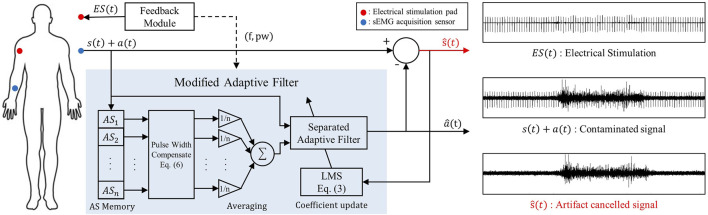
System block for artifact cancellation.

In this paper, an adaptive filter based on Least Mean Square (LMS) (Widrow and Hoff, 1988) is modified. Figure 2 shows the overall structure of the system, with the most crucial process being the calculation of the estimated noise $\left(\hat{a}\right)$ from the reference signal (y) through the adaptive Filter $\left(B\in R^{1\times n}\right)$ , which is shown in Formula 1. The calculated $\hat{a}$ is then subtracted from the input signal (x) as shown in Equation 2 to create the assumed signal $\left(\hat{s}\right)$ . The process by which the assumed signal $\hat{s}$ becomes similar to the desired signal s through the previously mentioned calculation is related to the update of B , the coefficient of the adaptive filter. The LMS method, one of several algorithms used for coefficient update of adaptive filters, is computed as Equation 3 through the Wiener-Hoff equation (Bueckner and Center, 1961). This is a method that updates B to minimize e by using the assumption of setting a reference signal with a low correlation with the desired signal and a high correlation with noise. Therefore, when using an adaptive filter based on LMS, selecting the correct reference signal is important. To adhere to the principles of correlation, we implemented three key modifications. First, based on the knowledge that sEMG has zero-mean gaussian distribution, we utilized multiple past artifacts to restore artifacts well (Sections 2.3–4). Second, we designed the system to cancel artifacts from frequency/pulse width varying feedback (Section 2.5). Finally, we separate artifact residual time range to selectively cancel and rapid convergence of filter coefficients.

$$\hat{a}\left(t\right)=B^{T}\left(t\right)\cdot Y\left(t\right)=\overset{n-1}{\underset{i=0}{\sum }}b_{i}\left(t\right)\times y\left(t-i\right)$$ (1)

$$\hat{s}\left(t\right)=x\left(t\right)-\hat{a}\left(t\right)=s\left(t\right)+a\left(t\right)-\hat{a}\left(t\right)≃s\left(t\right)$$ (2)

$$B\left(t+1\right)=B\left(t\right)+2\mu \cdot \hat{s}\left(t\right)\cdot Y\left(t\right)$$ (3)

### 2.3 Delayed references from sEMG signals

As mentioned in the previous section, the most critical aspect of using an adaptive filter based on LMS is determining the reference signal. In this study, to select a signal with a higher correlation to noise, the past artifacts of the same channel was stored and used as a reference signal. This approach ensures that signals measured in the same channel will produce similar artifacts compared to artifacts that appear in other channels. This is because the system between TENS and artifacts changes depending on channel location. The delayed artifact mentioned in the above process is stored during the residual time mentioned in Section 2.1 and used later, one entire artifact stored in this way is called an artifact sequence $\left(AS\in R^{1\times r}\right)$ .

### 2.4 Multi artifact sequences for reference signal

If the reference signal is as mentioned in Section 2.3, it includes both s(t) and a(t) components. In other words, this signal simultaneously contains the muscle signal and the artifact component to be removed from the input. To reduce performance degradation due to this, rather than using a single AS as a reference signal, the average of n AS was used as a reference signal. For this purpose, AS were stored in the system as shown in the lower left corner of Figure 2. The assumption of the above method is that the sEMG signal has a mean of 0 and follows a Gaussian distribution. This means that even if the muscles are activated at the same level, the raw sEMG signal does not have a constant level proportional to it. This leads that if n random sEMG signals are added, the probability that this sum will be zero increases significantly as n increases. For the result, the sum of n AS will effectively restore the shape of the artifact as shown in Formulas 4, 5 by deleting muscle signals.

$$if\;s∼N\left(0,\sigma^{2}\right),n\to \infty \Rightarrow E\left[s\right]=0$$ (4)

$$\overset{n}{\underset{i=1}{\sum }}x\left(i\right)=\overset{n}{\underset{i=1}{\sum }}s\left(i\right)+\overset{n}{\underset{i=1}{\sum }}a\left(i\right)≃\overset{n}{\underset{i=1}{\sum }}a\left(i\right),$$ (5)

### 2.5 Varying pulse width compensation

In order to utilize a sensory feedback system through TENS, various information is provided to the user using multiple frequencies and PW of TENS. Because these parameters are controlled by the system, they are prior information known before the artifact occurs. Since the amplitude of the artifact increases with increasing PW, compensation can be applied to make the adaptive filter robust against varying PW. For this purpose, a specific AS and the PW were stored together, and the previous AS was adjusted using the PW of the currently authorized TENS, as shown in Equation 6. In this study, an α value was empirically set in the range of 100–150.

$${AS}_{\mathrm{pw2}}^{\prime }={AS}_{\mathrm{pw1}}\times \frac{pw2+\alpha }{pw1+\alpha }$$ (6)

### 2.6 Filter separation for varying frequency and update phase

The frequency of TENS is controlled by an MCU internal timer, ensuring precise operation at precise time intervals. Consequently, filter can receive frequency information from the TENS system and predict the starting point before artifacts occur. In other words, the filter can distinguish whether input sample is in the artifact residual time range or not (1<k<r) . Through this separation, cancellation can be performed only when artifacts occur, regardless of the frequency of TENS. Also, the filter coefficient B is updated only during the residual time, facilitating relatively rapid convergence of the filter coefficient. During the residual time, the reference signal is calculated as in Equation 7 using the previous AS, and then the AS is updated as in Equation 8.

$$y\left(t\right)=\frac{1}{n}\overset{n}{\underset{i=1}{\sum }}\left[{AS}_{i}\left(k\right)\times \frac{pw+\alpha }{pw_{i}+\alpha }\right]$$ (7)

$$\left\{\begin{array}{c} {AS}_{i+1}\left(k\right)={AS}_{i}\left(k\right),\;i=1,\ldots ,n-1\\ {AS}_{1}\left(k\right)=x\left(t\right) \end{array}\right.$$ (8)

## 3 Experimental setup

### 3.1 Placements for sEMG acquisition and stimulation

**FIGURE 3:**
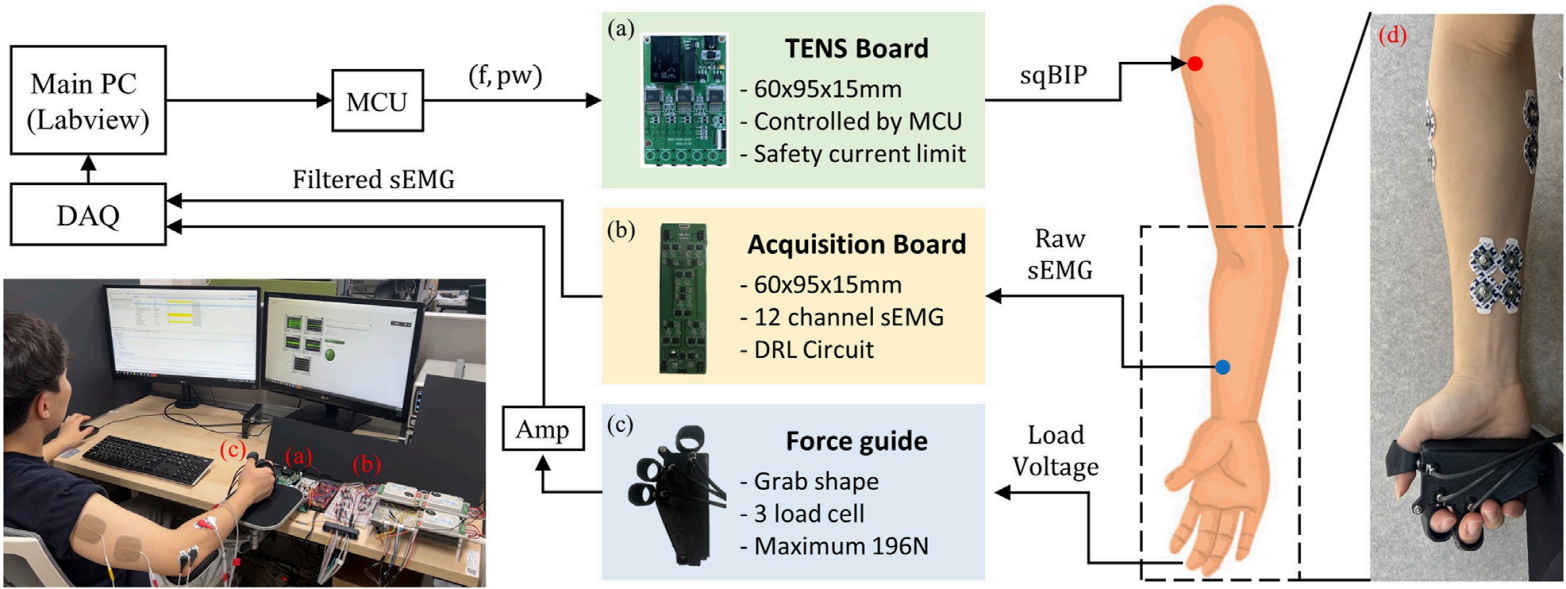
Experiment setup for subject and system configuration. **(A)** Developed TENS board circuit based on switching circuit and Howland current pump **(B)** sEMG acquisition board with DRL circuit **(C)** Force guide to maintaining finger force **(D)** Four sensor placement in forearm.

The placement of sEMG sensors and TENS pads is illustrated in Figure 3. Each sEMG sensor comprises two wet-type electrodes (Kendall, Covidien, Ireland). Two sensors are attached near the wrist, targeting the finger flexor muscles, while another two are positioned above the elbow, targeting both the wrist flexor and extensor muscles (Figure 3D). This configuration not only validates the robustness of the proposed method for various electrode positions but also allows for the independent utilization of two muscles in the forthcoming experiments. The remaining two electrodes are attached to the elbow, and all electrodes are connected to a self-developed signal acquisition board, incorporating signal filters and common noise removal.

For TENS, CWN2505 pads (CQMS Co., South Korea, square shape, 50 × 50 mm) commonly used in physical therapy were employed. Two pads deliver a minimal current to provide sensory input. Pads are attached between the biceps and triceps to ensure the transmission of electrical stimulation through the high radial nerve. TENS is delivered through the developed TENS board (Cho et al., 2023), featuring a voltage divider, DC converter, and the generation of square biphasic pulses determined by the input frequency and PW.

### 3.2 System communication configuration

The system configuration is established as illustrated in Figure 3, and all experiments are conducted with the main PC serving as the central hub. Communication from the main PC to the microcontroller (TMS320F28379D, Texas Instruments, United States) involves essential variables of electrical stimulation, such as frequency and PW, following various FS. The microcontroller communicates these variables to the TENS board (Figure 3A), which controls TENS through pads attached to the human skin. The sEMG signals are filtered by a signal acquisition board (Figure 3B) and transmitted to the main PC via the DAQ board (PCIe-6363, National Instruments, United States). We designed ergonomically shaped hardware for measuring the flexion force of three fingers (Figure 3C). This design includes the attachment of load cells (CBFSC-10, CASKOREA) to the tips of each finger, enabling the measurement of force when the user naturally clenches their fist. Subsequently, the output from each load cell undergoes amplification before being transmitted to the main PC through the same DAQ board.

## 4 Experimental protocol

### 4.1 Subject

Twelve healthy participants (aged 23–29 years) were recruited in this experimental study. None of the participants had a history related to any neurological or skin diseases. We gave a sufficient explanation about the experimental protocols and precautions, and participants filled out a consent form. Experimental protocols were approved by the KAIST Institutional Review Board (KAIST IRB No.KH 2023-231).

### 4.2 Threshold and load cell preparation

The threshold of sensation varies among individuals and can be influenced by the positioning of the TENS pads. Therefore, it is crucial to assess the boundary for the parameters of TENS such as PW before its application. Due to the utilization of a fixed amplitude for TENS, the PW is the most significant parameter affecting the subjects’ sensations. If the PW is too short, the subjects may not recognize that they are receiving TENS. On the other hand, if the PW is too long, it can cause muscle tremors or spasms, leading to discomfort. To search those boundaries, the PW was increased by 10 us in each step to measure both the Sense Threshold (ST) , where the subjects initially perceive the TENS, and the Pain Threshold (PT) , corresponding to the threshold at which the TENS becomes discomforting. The measured values were applied to the subjects, with the exclusion of the lower and upper 20 % of the safety range for stability in the experiment. Since there are also differences in grip strength among participants, we calibrated the measurement range of the load cells and normalized the measured values. Through adjustment of the gain in the load cell amplifier, the force sensor registers zero when participants exert no force and reaches one when 70 % of the maximum voluntary contraction (MVC) is applied. This ensures that participants consistently exert a similar level of force, thereby enhancing the accuracy and reliability of the experiment. Each procedure was conducted with participants in a comfortable seated position, their arms elevated and supported on a desk while maintaining a natural grip on the force sensor placed on the support.

### 4.3 Artifact cancellation in various feedback scenarios

**FIGURE 4:**
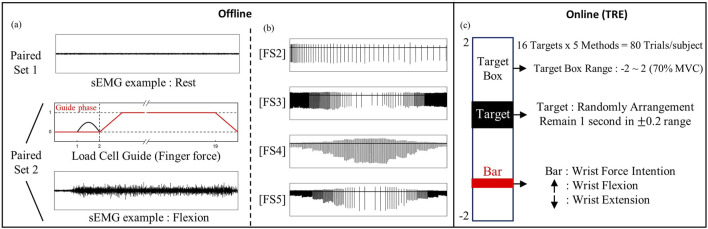
Offline/Online experiment protocol for validation **(A)** Paired set 1/2 experiment for rest/flexion. Load cell guide is given in visual feedback. **(B)** sEMG (including artifact) variation from different feedback scenarios, **(C)** TRE configuration for real-time validation.

This experiment aimed to evaluate the efficacy of the proposed method alongside other approaches for artifact removal in various FS. Each experiment for all conditions and models involves a paired set recording, with subjects either at rest or flexing their fingers. Figure 4A explains the paired set recording. Both recording had a duration of 20 s. In the rest recording (Paired set 1), subjects conducted the experiment with their arm and fingers comfortably relaxed. In the flexing recording (Paired set 2), a virtual load cell guide was provided to the user. The period from 2 to 19 s represents the signal acquisition phase. The time before and after this phase serves as the guide phase. In the initial guide phase, the monitor provides a visual cue signaling the start of the acquisition phase for the subject. Subsequently, within the next second, a linearly increasing guide, ranging from 0 to 1 in the force sensor’s monitor, is presented to instruct the subject to gradually clench their fist to a specific force level (70 % MVC, value: 1.0). Subjects maintained this force level with the visual clue in the acquisition phase (2–19 s) and released the force in the last second. All subjects sufficiently practiced to adeptly increase and sustain forces in both phases before the data acquisition.

The paired set recordings were performed in multiple trials introducing diverse scenarios with different frequencies and PW to validate the consistent removal of artifacts across various conditions. A total of five FS were tested, encompassing FS1) no stimulation, FS2) frequency variation: continuous increase of TENS period from the minimum period of 0.01 s, FS3) frequency variation: seven frequencies within the range typically perceptible by humans, FS4) PW variation: seven different PW within range from ST to PT, FS5) frequency and PW variation: combined FS3 and FS4 (Figure 4B). Seven frequencies were selected based on the types of sensations that humans can distinguish below 100 Hz. Ten recordings, via two types of paired set and five types of FS, were repeated once for each participant within the same order: paired set 1 (without TENS), FS1 to FS5, paired set 2 (with TENS), FS1 to FS5. Minimum 1 min rest is given between every recording.

### 4.4 Cancellation from simulated sEMG signal

To verify the two methodologies-use of multiple AS and PW compensation, a simulation test in which ground truth exists was conducted using signals acquired from the subjects. FS1 (without stimulation) while flexing finger was assumed to be a raw sEMG signal. In addition, other four FS while resting as assumed to be a simulated artifact. FS2 and FS3 were applied by changing the number of AS in the proposed method (Section 2.3). FS4 and FS5, in which PW was changed while recording, were designed to compare performance difference between with/without applying (Formula 6) in Section 2.4.

### 4.5 Real-time control experiment

We implemented the Target-reaching Experiment (TRE) which is a real-time control simulator with visual feedback (Figure 4C). It was referred from (Cho and Kim, 2022) and implemented in the Labview environment for all models to be compared. This experiment involves manipulating a bar based on measured sEMG values, aiming to precisely control its movement towards an arbitrary target. We utilized sEMG signals obtained during the wrist flexion and extension. Due to its opposing movements controlled by the two muscles, when one muscle is activated, the opposing muscle remains nearly inactive, creating an independent muscle activity highly correlated with wrist intention. To address the perturbation of the raw sEMG signals and use it as a control input, a second-order Butterworth low-pass filter (1 Hz cutoff frequency) was applied to get the envelope of the sEMG. The motion range of the bar was configured to span from a maximum of 2 in the wrist flexion direction to a minimum of −2 in the extension direction. The experiment comprised sixteen tasks, with target positions ranging from 1.7 to −1.7 in 0.2 increments, excluding 0.1 and −0.1. Success was defined as the bar remaining within a 0.2 range above and below the target value for 0.5 s. Any deviation beyond this range over 10 s was considered a failure. This experimental setup aims to evaluate control performance using sEMG signals after the cancellation of artifacts. Throughout this Experiment, the frequency and PW of TENS were randomly given. Selected frequency and TENS was same with the selection in Section 4.3.

Before the experiment began, participants were given sufficient time to practice the TRE test in without feedback environment to become familiar with the task. However, to minimize instances where participants might adapt and solve problems despite the presence of artifacts, no practice time was given during the feedback conditions. A total of five methods were implemented in the following order: 1) Without feedback/With feedback-2) Non Cancellation, 3) Proposed, 4) RE, and 5) FB. Each method was carried out in a randomized sequence for the mentioned 16 tasks. Five minute break was provided between each method to allow participants to rest and prevent sensory adaptation.

### 4.6 Performance indices

#### 4.6.1 Verify delayed signal and pulse width modulation

The preceding simulated sEMG signal is a process to compare the artifact removal performance according to various delays and the presence or absence of PW compensation. Unlike the actual situation where the sEMG signal and artifact are acquired simultaneously, there is a ground truth (raw sEMG signal). In other words, to compare artifact removal performance, the difference between the filtered result and the ground truth can be compared, and in this study, normalized root mean square error (NRMSE) was used.

#### 4.6.2 Artifact cancellation performance

In the process of acquiring sEMG signals, physically separating muscle signals from artifacts is inherently challenging, making the definition of ground truth difficult for cancellation performance validation. To assess the performance comparison, the SNR serves as a quantitative metric. Qualitative evaluations are also conducted by demonstrating results in both the time and frequency domains to show the precise recovery of signals.

SNR is computed using the rest condition of each set as intrinsic noise and the force condition as the signal. The presence of artifacts causes an increase in the magnitude of both the signal and noise. Consequently, artifact is expected to result in a decrease in the SNR value. Therefore, we can observe variations in the SNR value increase, depending on how well artifact cancellation methods perform, including both the proposed method and the comparison methods. When the filters operate effectively, the SNR value can generally be restored to the level of the non-stimulation condition. Moreover, within the same force condition for each channel, we expect diverse levels of muscle signals. Consequently, the SNR increase will be evaluated for each channel independently.

#### 4.6.3 Indices of the real-time control simulation

We employed four performance indices to compare online performances in the TREs, as referenced in Cho and Kim (2022). The selected indices include completion rate, completion time, trajectory efficiency, and overshoot. Trajectory efficiency is defined as the ratio between the actual trajectory length and the optimal length, represented by the direct linear distance from the initial to the end position. This measure serves as an indicator of signal stability while subjects control the bars in the TRE. Also the overshoot is the number that pass through the target.

## 5 Results

### 5.1 Restoring simulated signals

**FIGURE 5:**
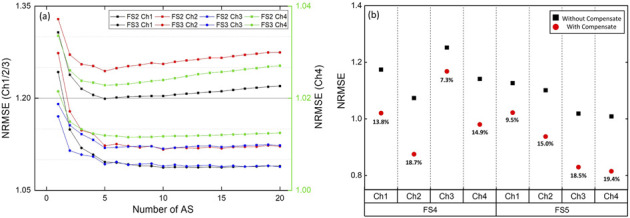
NRMSE difference according to proposed method **(A)** NRMSE difference by number of AS. Channel 1/2/3 use left y-axis and Channel 4 use right y-axis, **(B)** NRMSE difference for with/without PW compensate, decreased ratio is shown.

In the simulation experiment, the NRMSE value between the raw sEMG signal and the restored signal was calculated for each variable. Figure 5A shows the NRMSE calculated based on the delay in FS2 and FS3 scenarios, while Figure 5B illustrates the change in NRMSE with the presence or without PW compensate. Both graphs show the average values of 10 subjects, excluding the maximum and minimum values for each experiment and channel. The results of NRMSE showed a general trend of decreasing NRMSE with an increasing number of AS. However, the results were nonlinear, and there were cases where NRMSE increased as the number of AS became higher. This is expected to be due to reasons such as changes in posture over time. When performing one-way repeated ANOVA according to the number of AS, the most statistically significant difference from the case using one AS was generally distributed between five and ten, and also the largest NRMSE decrease was calculated at 10 As (6.57 % ). Afterward, ten AS were used in this study. In the case of PW compensation, applying the proposed method results in a decrease in NRMSE ranging from a minimum of 7.3 % to a maximum of 19.4 % across each experiment and channel. This confirms that using PW compensation in the proposed method helps to remove artifacts in situations where PW changes.

### 5.2 Artifact cancellation from sEMG signal

**FIGURE 6:**
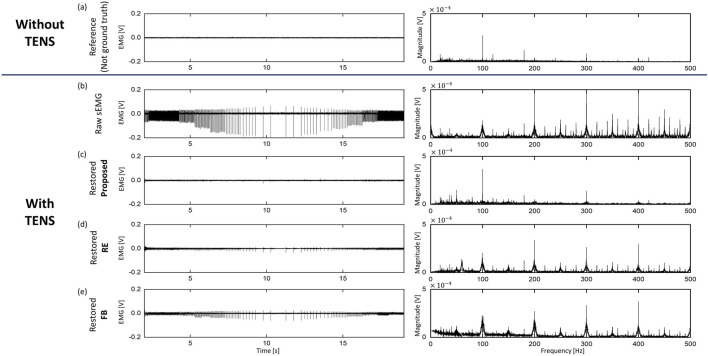
sEMG signals of Paired Set 1, FS5 from one participant. Every signals were expressed in both time/frequency domain. **(A)** sEMG signal without TENS (reference), note that this is not Ground Truth, **(B)** Raw sEMG signal with TENS, **(C–E)** restored sEMG signal from three different method.

**FIGURE 7:**
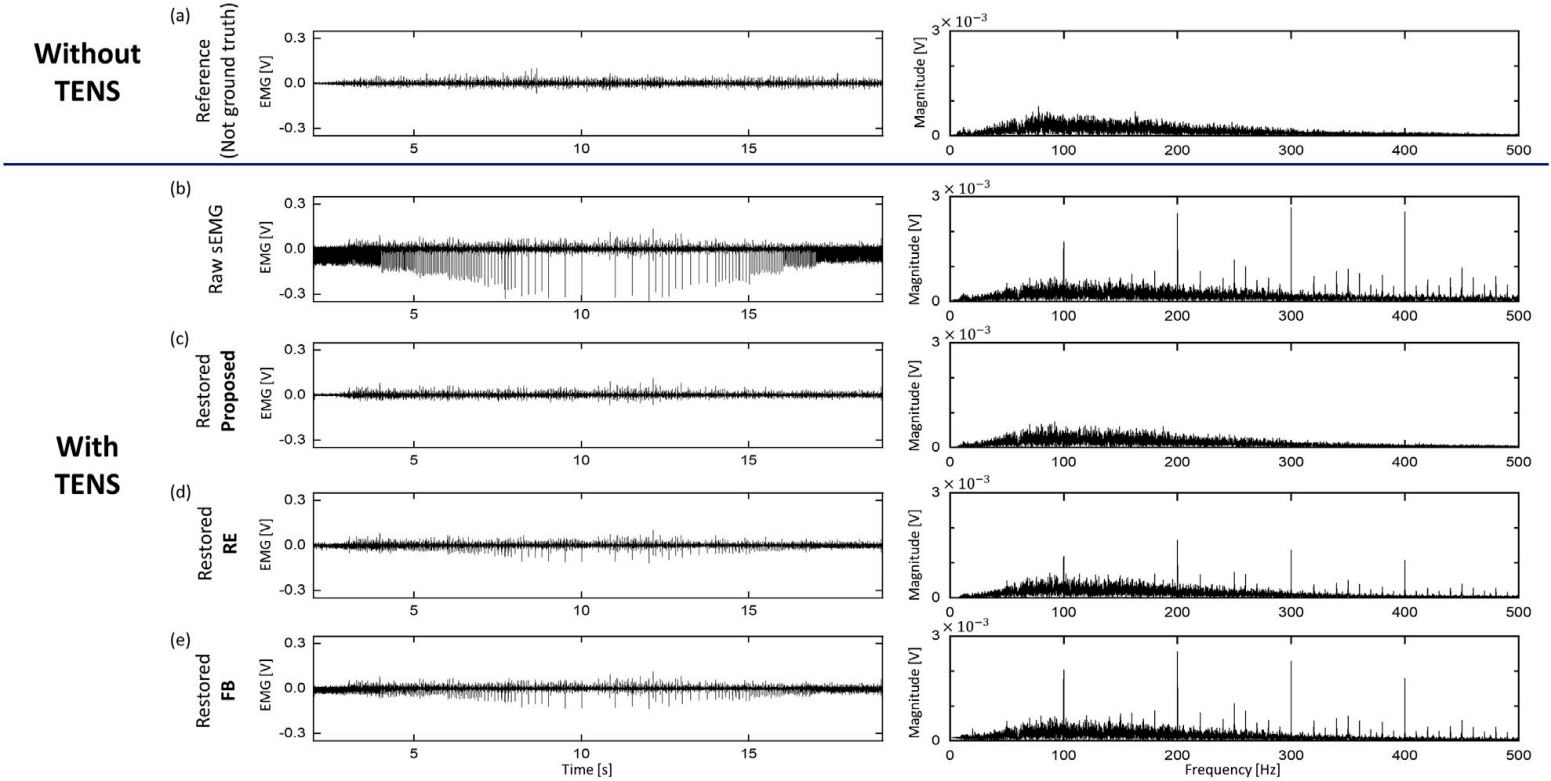
sEMG signals of Paired Set 2, FS5 from one participant. Every signals were expressed in both time/frequency domain. **(A)** sEMG signal without TENS (reference), note that this is not Ground Truth, **(B)** Raw sEMG signal with TENS, **(C–E)** restored sEMG signal from three different method.

Figures 6, 7 presents the results of offline signal processing acquired from channel 4 of one of the subjects. The horizontal axis is divided into paired sets, with each set displaying both time-domain and frequency-domain signals simultaneously. The top of the vertical axis represents FS1 (Without TENS). However, as this signal doesn’t precisely match the signal obtained below, it serves as a standard reference rather than the ground truth. The second row on the vertical axis is the raw sEMG acquired during FS5. The graphs below show the results of processing raw sEMG offline through the proposed method, RE method, and FB method, respectively. This graph is presented to qualitatively confirm the performance of each method, and it is noticeable that the level of artifacts is significantly reduced in the time domain. In addition, irregular bursts occur due to changes in frequency, but it was confirmed that they decreased to the reference signal level after applying the proposed method. On the other hand, it can be seen that bursts remain in the RE and FB methods.

**FIGURE 8:**
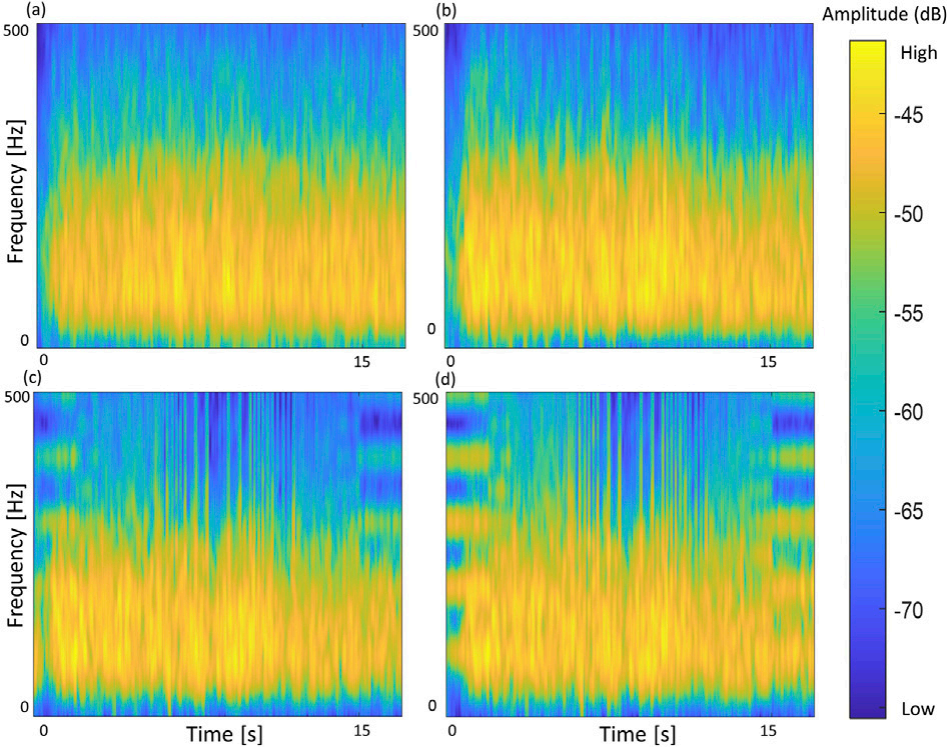
Power spectrum after artifact cancellation by each method. The original time domain signal is from Figure 7 paired set 2. **(A)** Without TENS (Reference), **(B)** Proposed, **(C)** RE, **(D)** FB.

Figure 8 is a time-frequency plot of the raw sEMG signal during flexion situation and the signal was removed in three ways (see Figure 7). This serves as a qualitative assessment of signal restoration. The reference signal and the proposed method show similar patterns across all sections. However, in the RE and FB methods where artifacts remain, 1) 100 Hz harmonics appear at the beginning and end sections where 100 Hz artifacts occur, and 2) blue vertical patterns of low-frequency artifacts were presented due to the elimination of high-frequency components. Since the proposed method uses past five AS as references for the LMS filter, signal delay may occur. This latency is directly influenced by the frequency of the TENS. However, it is important to note that sensory feedback typically involves high-frequency stimulation, which the human body perceives as vibration (Ara et al., 2014). In general, this frequency range falls between 50 and 100 Hz (10 20 ms), which leads to an approximate delay of 50,100 ms. This level of delay is considered manageable for real-time operation, and the results presented in Figures 6–8 support this claim.

### 5.3 SNR improvement via artifact cancellation methods

**FIGURE 9:**
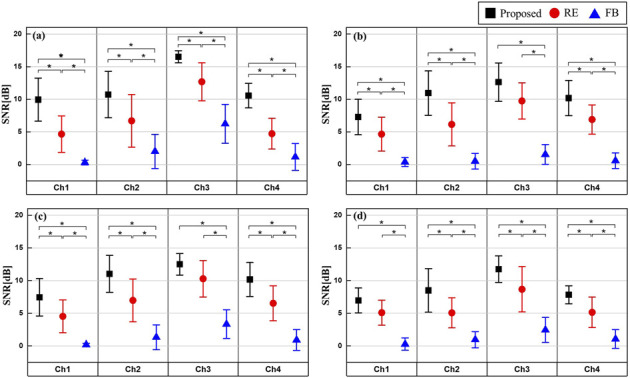
SNR increase after artifact cancellation by each feedback scenarios **(A)** FS2: Continuous Frequency Modulation, **(B)** FS3: Discrete frequency modulation, **(C)** FS4: Pulse width modulation, **(D)** FS5: Frequency and pulse width modulation.

TENS generates artifacts in the signal and noise of the paired set at similar levels, leading to a decrease in SNR. Figure 9 shows the average and standard deviation of the increased SNR when applying the Proposed, RE, and FB methods. These results are derived from data obtained from 10 out of the 12 subjects, excluding the minimum and maximum values. After confirming that there was a statistical difference in the increase in SNR for each method using one-way repeated ANOVA, the statistical difference for each method was verified using the Tukey-Kramer *post hoc* test. As a result, the FB method showed significantly lower performance across all channels in all experiments (*p* < 0.05). When comparing the proposed and RE methods, Ch3 of FS2, Ch3 of FS3, and Ch1 of FS4 didn’t reach statistical significance (*p* = 0.0529, *p* = 0.1145, *p* = 0.0576), but overall, the proposed method showed greater performance.

### 5.4 Target reaching experiment results

**FIGURE 10:**
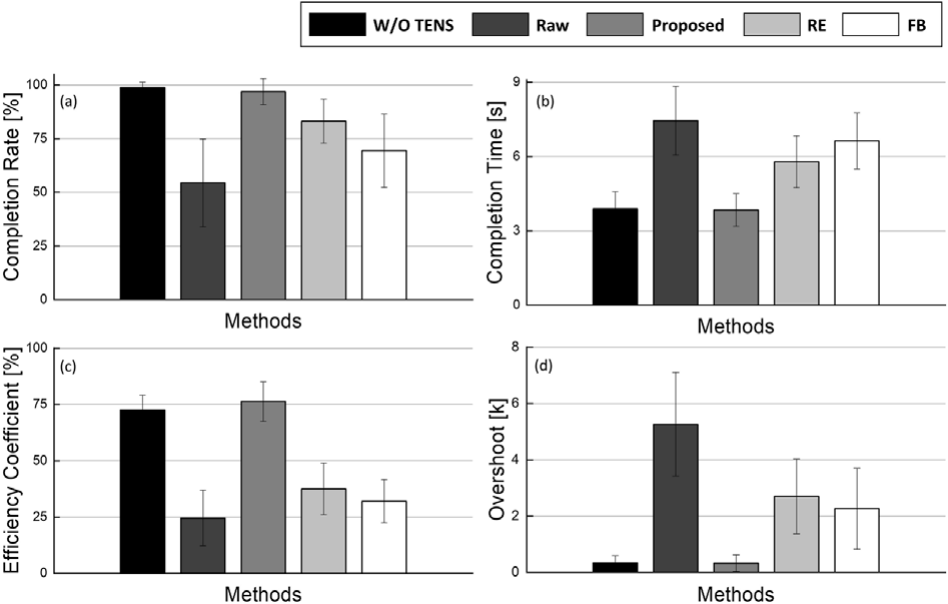
TRE performance indices result. **(A)** Completion rate **(B)** Completion time **(C)** Efficiency coefficient **(D)** Overshoot. *p*-value of without TENS and proposed method for each indices are *p* = 0.976, *p* > 0.99, *p* = 0.918, *p* = 0.938.

The results of TRE are shown in Figure 10. Since the leftmost bar represents the TRE result without TENS, it serves as a measure of the achievement goal. The proposed method shows the best results across all categories and reaches closely to the achievement goal. The mean and standard deviation of W/O TENS and proposed method are 98.75±2.64% , 96.88±6.07% in success rate, 3.88±0.70s , 3.84±0.66s in success time. Similar values were recorded in efficiency coefficient about 72.62±6.50% , 76.33±8.78% and in overshoot about 0.34±0.25 and 0.33±0.30 . For RE and FB methods, the values were noticeably increased compared to the situation where artifacts were not removed, although some differences were observed. In the case of RE, which showed better performance, there was a statistically significant difference in the remaining three categories compared to the scenario without TENS, except for the success rate category.

## 6 Discussion

### 6.1 Adaptive filter using for varying artifact cancellation

This study aims to remove artifacts measured along with sEMG signals when TENS is applied to users to provide sensory feedback. Also, method and evaluations are conducted to highly focused on the applicability to actual prosthetic system, rather than simply evaluating the performance of artifact removal. For this purpose, an adaptive filter was utilized, and a reference signal was employed by modifying the system to accurately remove artifacts while preserving the sEMG signal. In addition, we developed a system capable of preemptively responding to changing artifacts by using primary information such as frequency and PW. The circuit and system that implemented the proposed method had the following implications. First, there hasn’t been much research on artifact cancellation while stimulation are changing, which allows for the use of a variety of feedback techniques. These procedures will improve patients usability and are crucial technologies for closed-loop control of prosthetic hands. Also all designed circuits are small-sized and with low computational cost, proposed system can be implemented in prosthetic hand without modification. Moreover, an intention estimate online experiment was conducted into the experimental design and analysis to confirm real-time performance, and it verifies the feasibility that were not verified in previous methods. Although the real-time performance of the proposed method was validated through online experiments, proposed method introduces a delay of approximately 50–100 ms, which requires further quantitative analysis. Previous studies have reported methods with delays of 88/138 ms (Smith et al., 2011) and 100–125 ms (Farrell and Weir, 2007), which are similar to the delay observed in the proposed method. Moreover, since the method meets the commonly accepted threshold of 100 ms for real-time operation in the field of robotics, it can be considered sufficiently fast for real-time applications.

### 6.2 Comparison of other real-time approaches

The proposed method was compared with the RE and FB methods, which are two other methods that use adaptive filters. The comparison was conducted based on NRMSE in simulation scenarios, SNR improvement in real signals, and intention estimation in TRE experiments. As a result, we confirmed that the proposed method showed superior performance across most experimental scenarios and multiple electrode locations. Both validation were used in this procedure to improve reliability: a quantitative indicator and a qualitative way for verifying the real signals. This means that the artifact removal performance has been improved for scenarios involving varying TENS, and the method is robust across multiple electrodes. In TRE experiments, proposed method has does not show significant difference with non feedback, which allows us to speculate that it will have similar performance to other high-performance artifact cancellation methods. However, despite the comprehensive methodologies and experimental validations conducted to assess the performance of the proposed method, certain limitations remain. A notable limitation is the absence of additional quantitative metrics for evaluation. In this study, we aimed to remove artifacts from signals where sEMG and artifacts were measured concurrently, unlike previous research that often utilized synthetic signals composed of mixed sEMG and artifacts. Due to the lack of a definitive ground truth in this experimental setup, it is not feasible to accurately assess the separation error. Introducing a more reliable quantitative metric, beyond SNR, could enhance the robustness of our analysis.

In addition to the two comparison methods (RE, FB), Earley et al. (2022) proposed two artifact cancellation techniques: one using template subtraction and another employing the e-NLMS filter. Both methods demonstrated significant performance improvements after artifact removal, with the advantage of real-time implementation. While these methods share similarities with the approach presented in this paper, the proposed method offers several unique contributions. First, as mentioned by Earley et al., the LMS filter’s performance improves when the reference signal is highly correlated with the artifact. In this study, we utilized past artifacts to generate a reference signal that closely resembles the current artifact, specifically removing sEMG components. Furthermore, by including pulse width compensation, this method is expected to perform well in real-world applications that require various feedback. The two methods proposed by Earley et al. were validated offline using SNR and verified in both offline and online settings through pattern recognition accuracy. A detailed analysis of various parameters was conducted, with one of the most interesting thing is the analysis of the learning rate in template subtraction. As the range of past artifacts used increased, SNR/NRMSE initially improved but then showed a decline, a trend similar to the results depicted in Figure 5 of our work study regarding the optimal number of AS. These findings confirm the importance of appropriately selecting past artifacts for optimal performance.

### 6.3 Hyperparameter search

Optimization of several hyperparameters used in this study remains necessary. The convergence coefficient of the adaptive filters was empirically set to the same value across all comparison groups. For PW compensation, the α value was determined by inspecting the signal for each user; however, a comprehensive performance analysis was not conducted based on. Although α is not highly sensitive to the results of PW compensation, using an optimized value could enhance performance. Moreover, individual differences among users suggest that a personalized approach to α selection could further improve outcomes. Additionally, the simulation results were utilized to roughly determine the number of AS to be used as reference signals. We observed that the improvement in NRMSE diminished as the number of reference signals increased. However, this number may vary depending on the TENS frequency, and the values set in this paper can serve as a general guideline.

### 6.4 Limitation of additional artifacts

While we have demonstrated the effectiveness of the proposed adaptive filter for removing artifacts induced by TENS, the current study did not account for motion artifacts, which are prevalent in real-world applications (Nordin et al., 2018). Motion artifacts, caused by limb movements and electrode displacement, can significantly affect signal quality and pose a challenge for accurate artifact removal. The proposed method relies on a delayed signal as a reference for the adaptive process, which suggests the potential for motion artifact cancellation. However, further experimental validation across a range of scenarios is required to confirm its effectiveness in this context.

## 7 Conclusion

In this study, the modified adaptive filter, primarily for reference signal, was introduced and its performance was verified through both offline and online experiments. Multi-delayed sEMG signals and filter separation were used to improve filter performance, and the system utilized prior information to proactively respond to varying frequency and pulse width. The number of delayed signals and pulse width compensation performances are verified by simulated signals. Additionally, the overall performance of the proposed method was confirmed by SNR and reconfirmed through an intention estimation online experiment. Although the proposed method wasn’t verified for all scenarios, we demonstrated that applying it enables a closed-loop prosthetic hand system.

## Data Availability

The original contributions presented in the study are included in the article/Supplementary Material, further inquiries can be directed to the corresponding author.
